# Ultra-stable Electrochemical Sensor for Detection of Caffeic Acid Based on Platinum and Nickel Jagged-Like Nanowires

**DOI:** 10.1186/s11671-018-2839-0

**Published:** 2019-01-08

**Authors:** Jin Wang, Beibei Yang, Fei Gao, Pingping Song, Lei Li, Yangping Zhang, Cheng Lu, M. Cynthia Goh, Yukou Du

**Affiliations:** 1 College of Chemistry, Chemical Engineering and Materials Science, Soochow University, Suzhou, 215123 People’s Republic of China; 2 College of Biological, Chemical Sciences and Engineering, Jiaxing University, Jiaxing, Zhejiang 314001 People’s Republic of China; 3 Department of Chemistry, Department of Materials Science and Engineering, Institute of Medical Science, University of Toronto, Toronto, ON M5S 3H6 Canada

**Keywords:** PtNi jagged-like nanowires, Electrochemical sensor, Caffeic acid, Stability

## Abstract

Electrochemical sensors have the high sensitivity, fast response, and simple operation for applications in biological, medical, and chemical detection, but limited by the poor stability and high cost of the electrode materials. In this work, we used PtNi lagged-like nanowire for caffeic acid (CA) electrochemical detection. The removal of outer layer Ni during reaction process contributed to the rehabilitation of active Pt sites at the surface, leading to the excellent electrocatalytic behavior of CA sensing. Carbon-supported PtNi-modified glassy carbon electrode (PtNi/C electrode) showed a broad CA detecting range (from 0.75 to 591.783 μM), a low detection limit (0.5 μM), and excellent stability. The electrode preserved high electrocatalytic performance with 86.98% of the initial oxidation peak current retained after 4000 potential cycles in 0.5 mM caffeic acid solution. It also demonstrates excellent anti-interference capability and is ready for use in the real sample analysis.

**Electronic supplementary material:**

The online version of this article (10.1186/s11671-018-2839-0) contains supplementary material, which is available to authorized users.

## Background

Metal nanomaterials with specific structure, size, and shape have aroused tremendous attention in electrochemical field because of their remarkable electrocatalytic performance. Special designed metal electrocatalysts possess sufficient exposed active sites and specific facets in electrochemical reaction, resulting in an enhanced electrochemical activity, stability, and durability [1, 2]. Platinum (Pt) with diverse morphologies and high electrocatalytic activity has been the hotspot in electrochemical sensor, fuel cell, and supercapacitor [3–5]. However, Pt can be easily deactivated when adsorbing intermediates and/or reaction byproducts during the reaction process [6]. Meanwhile, the low durability and high price were also the obstacles that limit its utilization. To overcome these barriers, bimetallic Pt-(Ni, Cu, Sn, Pd) electrocatalysts were prepared to improve the Pt utilization and to enhance the long-term stability of the catalysts [7, 8].

Caffeic acid (CA, 3,4-dihydroxycinnamic acid), a kind of phenolic compound and the major hydroxycinnamic acid presented in human diet, is found in feverfew, coffee beans, teas, wines, and quite a few fruits [9]. CA is important in protecting the cells from induction of cell apoptosis, treating the therapy of leucopenia and thrombocytopenia in clinical diagnosis, and inhibiting the activity of phosphodiesterase (the major ingredient of snake venom) [10]. Furthermore, as the antioxidant, CA has been widely used in cosmetics, hair dyes, antibacterial agent, and anti-mutagen. Accordingly, it is vitally important to quantitatively detect CA in clinic, research, and the daily life. In the last few years, many techniques have been developed to detect CA. For instance, Cai et al. designed a fluorometric assay platform for fluorescence detection of CA [11], Khezeli et al. detected the CA by a green ultrasonic-assisted liquid–liquid microextraction based on deep eutectic solvent [12], Konar et al. measured the CA by supercritical fluid extraction [13], and Liu et al. detected the CA by electrochemical method with synthesized nanocomposite [14]. Among them, electrochemical detection has gained the most attention due to its stability, sensitivity, and fast response. A majority of reports are focused on carbon material-based nanocomposites, such as graphene-based Au nanoparticles and PEDOT (Au–PEDOT/RGO) [14], MnO_2_-embedded flower-like hierarchical porous carbon microspheres (MnO_2_/CM) [15], and gold/palladium nanoparticles decorated graphene flakes (Au/PdNPs-GRF) [10]. Compared with carbon-based system, Pt-based ones are expected to have even higher electrocatalytic sensitivity, but due to the abovementioned issues, it is seldom used toward CA sensing.

Inspired by this, we herein report a facile solvothermal method to synthesize a novel class of PtNi jagged-like nanowire, and investigate its application in electrochemical sensing toward CA. TEM and HAADF-STEM images showed the specific structure and morphology of the PtNi jagged-like nanowires. Electrochemical characterization revealed superior stability and high electrochemical activity of PtNi/C electrode, in which the Ni was dissolved from the outer part of PtNi jagged-like nanowire during the reaction process to expose more Pt active sites on the electrocatalyst, leading to an enhancement of stability and electrocatalytic activity [16]. The as-prepared electrocatalyst is superior than the commercial Pt/C catalyst in reactivity, suggesting a new direction for electrocatalytic in CA detection.

## Methods

### Reagents

Nickel (II) acetylacetonate (Ni (acac)_2_, 99%), platinum (II) acetylacetonate (Pt (acac)_2_, 97%) and glucose, and oleylamine (OAm, 70%) were all obtained from Sigma-Aldrich (Shanghai, China). CTAC (CH_3_(CH_2_)_15_N (Cl) (CH_3_)_3_, 99%) was bought from Aladdin. Commercial carbon powder and carbon-supported Pt catalyst (Pt/C, 20 wt% of Pt nanoparticles on carbon black) were purchased from Shanghai Hesen Electric Co., Ltd. Nafion solution (5 wt%) was bought from Alfa Aesar. Isopropanol, methanol, ethanol, and cyclohexane were supplied by Beijing Tongguang Fine Chemicals Company. All reagents were used without further purification, and all solutions were freshly prepared with double-distilled water. The Britton-Robinson (BR) buffer solutions were prepared from phosphoric acid, boric acid, and glacial acetic acid aqueous solutions (all solutions at concentration of 0.2 M) were used as the supporting electrolyte; the pH value of the final solutions in all experiments was adjusted with sodium hydroxide solution (0.1 M).

### Synthesis of PtNi Jagged-Like Nanowires

PtNi jagged-like nanowires were synthesized by a facile solvothermal method [17]. In details, Ni (acac)_2_ (2.1 mg), Pt (acac)_2_ (10 mg), glucose (30 mg), CTAC (60 mg), and 7.5 mL OAm were added into a three-neck flask. After ultrasonicating for half an hour, the three-neck flask was transferred to oil bath and kept at 180 °C for 10 h before cooling down to room temperature. The product was collected by centrifugation (10,000 rpm, 5 min) and washed four times with cyclohexane and ethanol, respectively. The as-obtained nanowires were dispersed in cyclohexane for further use.

### Preparation of Carbon-Supported PtNi Jagged-Like Nanowire Catalyst-Modified Electrode (PtNi/C/GCE)

The PtNi/C catalyst was prepared by mixing the carbon powder (9.5 mg), Nafion (20 μL), and the as-synthesized PtNi jagged-like nanowires (1 mL) in ethanol (9 mL). After ultrasonicating for 1 h, the resulting paste was collected in vial for further use. The working electrode was prepared by applying 0.48 μg PtNi/C paste on the cleaned GCE.

### Characterization

The morphology and microstructure of the sample were characterized by transmission electron microscopy (TEM), scanning transmission electron microscopy (STEM), and energy-dispersive X-ray spectroscopy (EDS) mapping on a FEI Tecnai F20 operating at 200 KV. XRD patterns of the PtNi jagged-like nanowires were acquired on an X’Pert-Pro MPD diffractometer (Netherlands PANalytical) equipped with a Cu Kα radiation. X-ray photoelectron spectroscopy (XPS) analysis was employed on a VG Scientific ESCALab 220XL electron spectrometer using 300 W Al Kα radiation.

### Electrochemical Measurements

The electrochemical measurements were carried on CHI760e electrochemical workstation (Chen Hua Instrumental Co., Ltd., Shanghai, China) with a standard three-electrode system. The platinum wire, glassy carbon electrode (GCE), and saturated calomel electrode (SCE) were used as the counter, working, and reference electrodes, respectively.

## Results and Discussion

### Sample Characterization

**Fig. 1:**
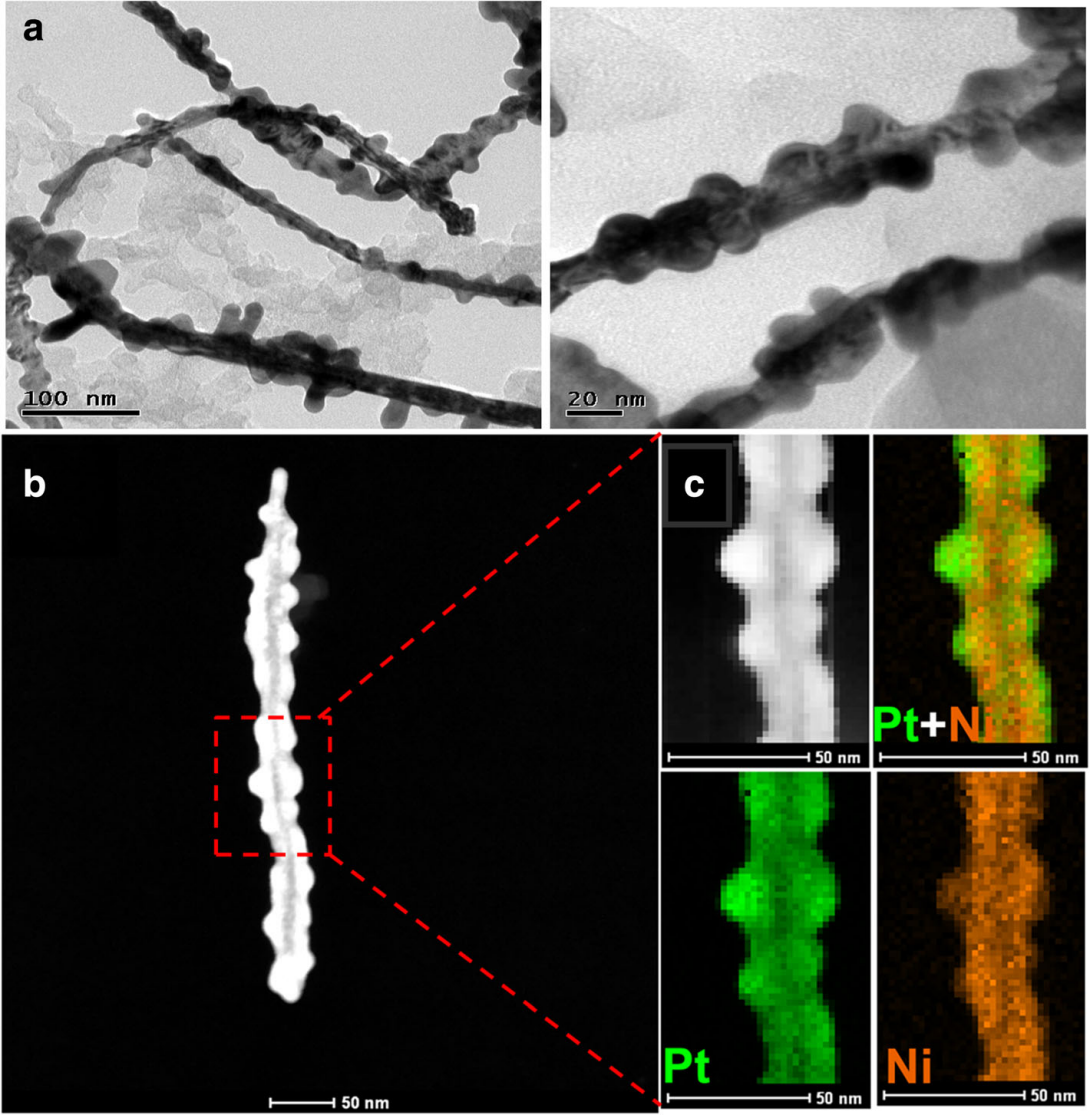
**a** TEM images, **b** HAADF-STEM images, and **c** STEM-EDS elemental mapping of Pt-Ni jagged-like nanowire

The morphology and surface structure of as-synthesized PtNi jagged-like nanowires sample was characterized by transmission electron microscopy (TEM) and high-angle annular dark-field scanning transmission electron microscopy (HAADF-STEM). Figure 1a exhibits the representative TEM images of the as-prepared, carbon-supported PtNi jagged-like nanowires, which show a predominantly jagged-nanowires morphology with an average width of 18 nm. The morphology of PtNi sample was further confirmed by HAADF-STEM and elemental mapping images. As shown in Fig. 1c, the evenly distributed Pt and Ni in the composition maps indicate that the as-prepared PtNi jagged-like nanowire is primarily consisted of PtNi alloy, with the jagged edge about 2–5 nm thick.

The as-prepared PtNi jagged-like nanowires possess the face-centered cubic (fcc) structure, as measured by the X-ray diffraction (XRD) pattern (Additional file 1: Figure S1). The main diffraction peaks of PtNi nanowires were situated between fcc Ni (JCPDS No. 650380) and fcc Pt (JCPDS No. 04-0802) demonstrating the formation of PtNi alloy. The sharp diffraction peaks located at 41.33, 48.06, 69.29, and 85.13° of 2*θ*, correspond to the typical fcc (111), (200), (220), and (311) planes of PtNi alloy, respectively [5]. X-ray photoelectron spectroscopy (XPS) was also performed to investigate the surface chemical composition of PtNi jagged-like nanowires. Additional file 1: Figure S2a shows the XPS spectra of Ni 2p deconvoluted into three peaks attributed to Ni 2p3/2, Ni 2p3/2, and Ni 2p1/2 species located at 855.1, 862.1, and 880.5 eV, demonstrating that portion of the surface Ni is in an oxidation state [1, 18]. Additional file 1: Figure S2b shows the distinct Pt 4f XPS patterns; the binding energies (BEs) of the doublet peaks located at 71.4 and 74.8 eV correspond to the Pt 4f7/2 and Pt 4f5/2, confirming the formation of metallic Pt. Meanwhile, the Pt 4f7/2 BE had a positive shift comparing with the pure metallic Pt, which was mainly due to the electron donation from Pt to Ni generating more electron deficient on the Pt atoms [8].

### Electrochemical Characterization of the PtNi Jagged-Like Nanowire-Modified Electrode

**Fig. 2:**
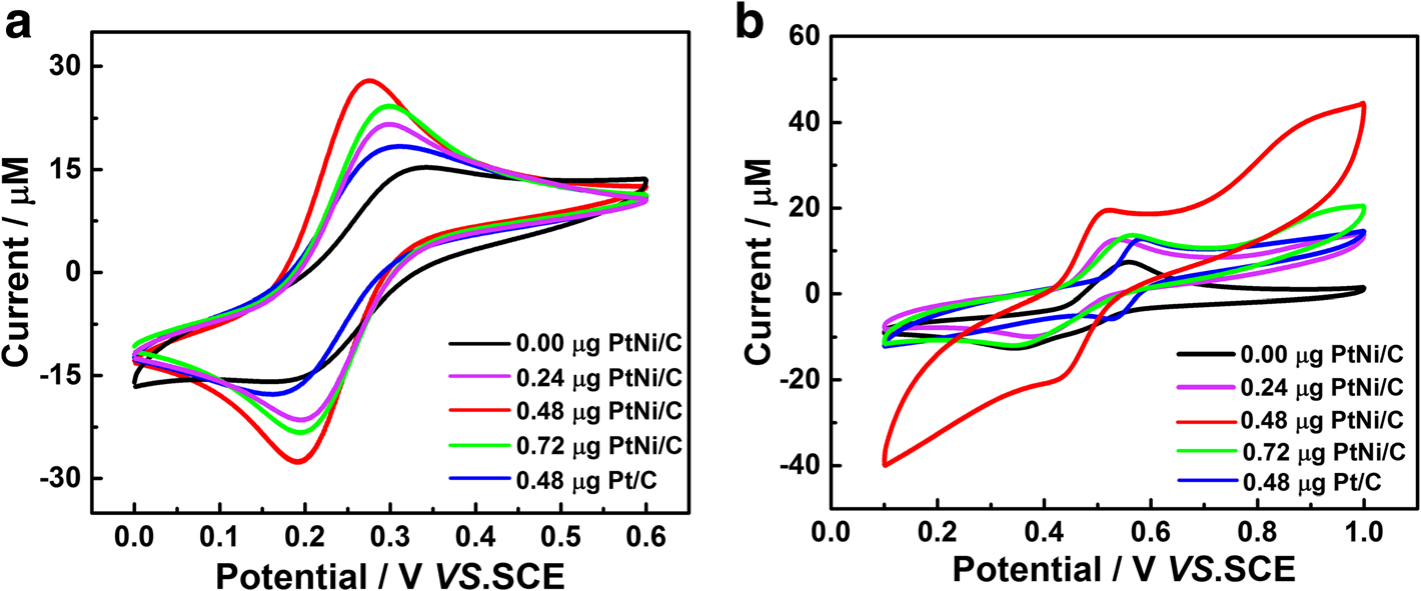
The CVs of 0 μg, 0.24 μg, 0.48 μg, and 0.72 μg PtNi/C and 0.48 μg Pt/C-modified GCE in 0.5 mM K_3_[Fe (CN)_6_]/ K_4_[Fe (CN)_6_] containing 0.1 M KCl (**a**) and in 0.1 M BR buffer solution (pH = 2.0) containing 0.5 mM CA (**b**) at a scan rate of 100 mV s^−1^

The electrochemical performance of different amount (0 μg, 0.24 μg, 0.48 μg, and 0.72 μg) of PtNi/C and 0.48 μg commercial Pt/C catalyst-modified electrodes was explored by CV scan in 0.5 mM K_3_[Fe (CN)_6_]/ K_4_[Fe (CN)_6_] solution containing 0.1 M KCl at a scan rate of 100 mV s^−1^, and the result is shown in Fig. 2a. As indicated in Fig. 2a, 0.48-μg PtNi/C-modified electrode shows the highest current and narrowest peak-to-peak potential separation (∆Ep), indicating 0.48-μg catalyst loading is the optimum choice to facilitate that can accelerate electron transfer between the PtNi/C electrode and electrolyte solution. At the same time, the influences of the four different amount of the PtNi/C and commercial Pt/C electrocatalyst loading in CA detection were also investigated (Fig. 2b). Obviously, the 0.48-μg PtNi/C-modified electrode owns the best electrocatalytic activity in CA oxidation when comparing with the other electrodes. Such an increase of current mainly ascribed to the following reasons, firstly, overloaded PtNi jagged-like nanowires will lead to an accumulation of catalyst, which blocks the majority of activity sites buried inside and hiders the electron transfer accordingly. On the other hand, insufficient catalyst cannot offer enough activity sites for CA oxidation, thus resulting in a poor electrocatalytic activity.

### Working Mechanism of PtNi/C Electrode in CA Detection

It is well known that pH value will affect the electrocatalytic activity in the electrochemical redox process. Therefore, the influence of pH on the CA electrochemical sensing was studied based on PtNi/C electrode. As displayed in Additional file 1: Figure S3, the current increased when pH varied from 1.0 to 2.0, then decreased while pH ranges from 2.0 to 7.0; thus, pH 2.0 was selected as the optimal pH in the experiment. The redox potential of CA shifts negatively with the increase of pH value, indicating the protons were involved in the redox process. The linear equation was expressed as E_pa_ (V) = − 30.28 pH + 0.6145 (*R*^2^ = 0.9522) with a slope of 30.28 mV/pH, proving two electrons and two protons were involved in the CA oxidation based on Nernst equation [14]. The reaction mechanism was shown as following (Eq. 1): .

The reaction characters of CA on PtNi/C/GCE was recorded by CVs at various scan rates in BR buffer solution (pH = 2.0) containing 0.5 mM CA. As shown in Additional file 1: Figure S4, the redox peak currents increased linearly with the increased scan rate from 20 to 200 mVs^−1^, the linear regression equations were obtained as *I*_pa_ (μA) = 131.472 c (μM) + 5.858 (*R*^2^ = 0.997) and *I*_pc_ (μA) = − 152.189 c (μM) − 5.238 (*R*^2^ = 0.994), indicating a typical adsorption-controlled process. Furthermore, the redox peak potentials changed barely within the checked scan rate; therefore, the redox process of CA at PtNi/C/GCE can be considered to be a quasi-reversible process [19]. The electron transfer number (*n*) was obtained from the following equation: ∆*E* = 59/*n* [20], where ∆*E* (mV) is the difference of the oxidation peak potential and reduction peak potential of CA and it is calculated to be 24.3 mV. According to the equation, *n* is 2.4, which means two electrons are involved in the electrochemical reaction of CA, which matches well with the result of *E*_pa_ vs pH value.

### Determination of CA on PtNi/C-Modified Electrode

**Fig. 3:**
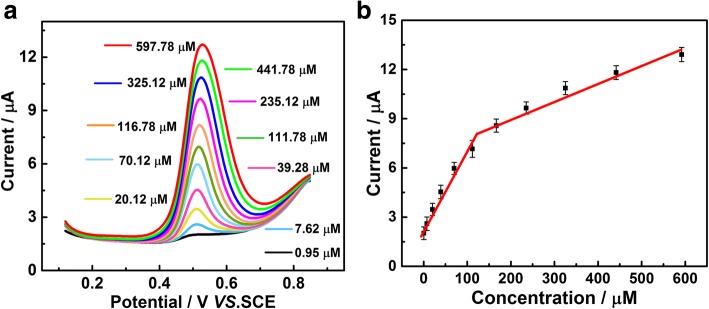
DPV of the 0.136 μg PtNi/C-modified electrode in 0.1 M BR buffer solution (pH = 2.0) solution containing different concentrations of CA (**a**) and the plots of oxidation currents versus the concentration of CA (**b**)

Differential pulse voltammetry (DPV) was commonly applied in quantitative detection of trace component due to its high sensitivity. In the DPV procedure, the current which was generated by impurities in redox reaction can be removed from the current differential reduction, leading to a higher sensitivity and low detection limit [21–24]. Figure 3a depicts the DPVs on PtNi/C-modified electrode in 0.1 M BR buffer solution (pH = 2.0) containing various concentrations of CA. An obvious enhancement of oxidation peak current was observed with the increase of the CA concentration. The oxidation peak current showed the good linear relationship versus the CA concentration and was expressed as *I*_pa_ = 0.0389 c + 2.59 (*R*^2^ = 0.95) and *I*_pa_ = 0.0107 c + 6.83 (*R*^2^ = 0.92) in the ranges of 0.75–111.783 μM and 111.783–591.783 μM, respectively. The detection limit is calculated to be 0.5 μM. The result is better than the previous reports (Additional file 1: Table S1) with different CA detecting methods. The two linear regression equations in Fig. 3b are related to different CA adsorption behaviors at different concentrations. At a relative low CA concentration, the rapid adsorption of CA on PtNi/C electrode leads to a quick increase of oxidization peak current. However, with the increase of CA concentration, excess CA, and impurities will be accumulated on the surface of PtNi/C electrode, resulting in a slow increase of oxidation peak current.

### Stability

**Fig. 4:**
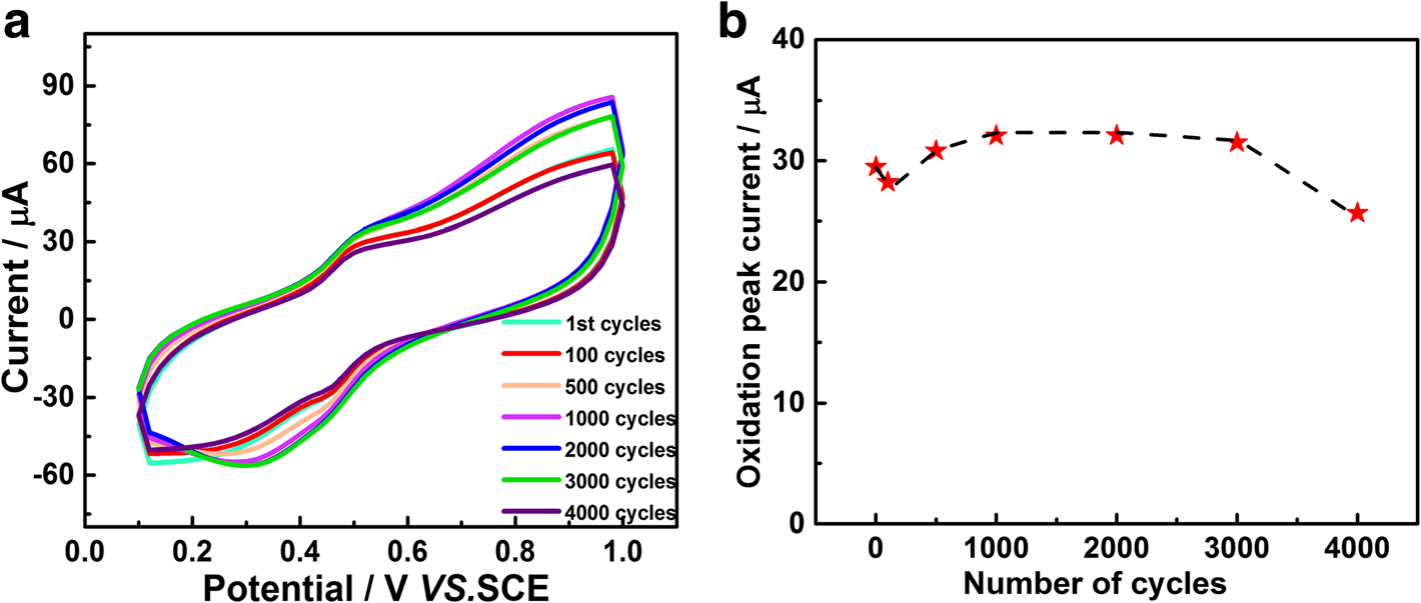
**a** The CVs of the 0.48 μg PtNi/C electrode in 0.5 mM CA after various numbers of potential cycles at a scan rate of 100 mV s^−1^. **b** The oxidation peak current vs number of cycles

Cyclic voltammetry was carried out to evaluate the stability of PtNi/C electrode by potential cycling. The voltammetric profiles of PtNi/C in 0.1 M BR buffer solution that contain 0.5 mM CA after 1, 100, 500, 1000, 2000, 3000, and 4000 cycles are shown in Fig. 4a. As can be seen, a decline of the oxidation peak current was observed at the initial 100 cycles, which is mainly because of the rapid absorption of the impurities on PtNi/C electrode surface that will cover the Pt active sites, resulting in a sluggish redox reaction of CA. Then, the oxidation peak current rose quickly from 100 to 1000 cycles. The current enhancement normally means the population of active sites (Pt) increase in the electrode. Normally, it decreased during cycling due to the surface passivation. The oxidation peak current drops slowly with further increasing the number of cycles, possibly due to the fact that more impurities were adsorbed on the PtNi/C electrode. After 4000 potential cycles, the oxidation peak current decreased to 13.02%, which is significantly better than commercial Pt/C electrode (Additional file 1: Figure S5). The surface morphology and structural information were checked by TEM, HAADF-STEM, and STEM-EDS elemental mapping analyzed (Additional file 1: Figure S6) to figure out the origin of active site variation during the cycling process. There was no obvious morphology change before (Fig. 1a) and after (Additional file 1: Figure S6a) cycling. Interestingly, STEM-EDS elemental mapping analyzed in Additional file 1: Figure S6c demonstrated that Ni was removed particularly in the jagged area, mainly because outer layer Ni was dissolved into the electrolyte. The removal of Ni rehabs the active Pt sites, slowing the surface passivation, leading to the increase of oxidization current and CA detection stability.

### Repeatability, Interference, and Real Sample Analysis of PtNi/C Electrode

To investigate the repeatability of PtNi/C electrode, ten parallel PtNi/C electrodes were used in 0.1 M BR buffer solution (pH = 2.0) containing 0.5 mM CA. The relative standard deviation (RSD) of oxidation peak current was found to be 3.7%, confirming that the as-prepared PtNi/C electrodes have good repeatability. The anti-interference capability of PtNi/C electrode was evaluated in 0.5 mM CA containing 100-fold concentration of tannic acid, resveratrol, tartaric acid, gallic acid, citric acid, P-coumaric acid, succinic acid, and malic acid. As shown in Additional file 1: Figure S7, the relative accuracy is between 97.9 and 105%, indicating the PtNi/C electrode owns glorious anti-interference performance. The real sample analysis of PtNi/C electrode for CA detection was monitored by using commercial red wine diluted 30 times with BR buffer solution (pH = 2.0). 50, 100, and 200 μM L^−1^ caffeic acid were added into the wine samples, respectively. The real sample analytical experiment was repeated for three times. The relative standard deviation and recovery information of the three samples in Additional file 1: Table S2 exhibited the very satisfactory results with the recovery rate ~ 100%. These results suggest the as-prepared PtNi/C electrodes are ready for CA detection in real samples in an effective and accurate manner.

## Conclusion

In summary, the novel PtNi jagged-like nanowires were prepared for CA sensing in this work. The PtNi electrocatalyst displayed superior stability by keeping 86.98% of the initial oxidation peak current after 4000 cycles. The exceptional stability is mainly ascribed to the rehabilitation of Pt active sites on the surface when Ni is removed during reaction. Moreover, the PtNi/C electrode also exhibited good electrochemical performance with a wide linear range of 0.75–591.78 μM and a low detection limit at 0.5 μM. Furthermore, the PtNi/C electrode showed satisfactory results in commercial wine detection. Our study may provide a valuable approach in CA electrochemical sensing by using Pt-based bimetallic system.

## Additional file

Additional file 1: **Figure S1.** XRD patterns of PtNi jagged-like nanowires. Figure S2 (a) Pt 4f and (b) Ni 2p deconvoluted XPS spectras of PtNi jagged-like nanowires. Figure S3 The CVs of 0.48 μg PtNi/C modified GCE in 0.1 M BR buffer solution containing 0.5 mM caffeic acid at pH ranging from 1.0 to 7.0 (a) and the plots of the anodic peak potential against pH (b). Figure S4 CVs of the 0.48 μg PtNi/C modified GCE in 0.1 M BR buffer solution (pH = 2.0) containing 0.5 mM caffeic acid at scan rates from 20 to 200 mV s^−1^ (a) and the plots of anodic and cathodic peak currents to the scan rates (b). Figure S5 The CVs of the 0.48 μg PtNi/C electrode (a) and Pt/C electrode (c) in 0.5 mM CA after various numbers of potential cycles (1–4000 cycles) at a scan rate of 100 mV s^−1^. The oxidation peak currents of 0.48 μg PtNi/C electrode (b) and Pt/C electrode (d) vs number of cycles. Figure S6 (a) TEM images, (b) HAADF-STEM images and (c) STEM-EDS elemental mapping of Pt-Ni jagged-like nanowires after subjecting the sample to potential cycling for 4000 cycles between 0.1–1.0 V in 0.1 M BR buffer solution electrolyte at 100 mV s^−1^. Figure S7 Relative analytical response (I_pa_/I_p_) of PtNi/C electrode in 0.1 M BR buffer solution (pH = 2.0) containing 0.5 mM caffeic acid in presence of different interfering species: 10 mM tannic acid, resveratrol, tartaric acid, gallic acid, citric acid, P-coumaric acid, succinic acid, and malic acid. Table S1 Comparison of the linear range and detection limit between the proposed method and other reported detection methods for caffeic acid. Table S2 Determination of CA in red wine samples (DOCX 2679 kb)

## Abbreviations

∆E: Difference of the oxidation peak potential and reduction peak potential.
CA: Caffeic acid
CV: Cyclic voltammetry
DPV: Differential pulse voltammetry
EDS: X-ray spectroscopy
GCE: Glassy carbon electrode
PtNi: Platinum and nickel
STEM: Scanning transmission electron microscopy
XPS: X-ray photoelectron spectroscopy
