# Supplementary material for: Ultra-stable Electrochemical Sensor for Detection of Caffeic Acid Based on Platinum and Nickel Jagged-Like Nanowires
Source: Nanoscale Res Lett. 2019 Jan 8;14:11. doi: 10.1186/s11671-018-2839-0 (PMC6325053; doi:10.1186/s11671-018-2839-0)
Supplement: Supplementary file 1 — Figure S1. XRD patterns of PtNi jagged-like nanowires. Figure S2 (a) Pt 4f and (b) Ni 2p deconvoluted XPS spectras of PtNi jagged-like nanowires. Figure S3 The CVs of 0.48 μg PtNi/C modified GCE in 0.1 M BR buffer solution containing 0.5 mM caffeic acid at pH ranging from 1.0 to 7.0 (a) and the plots of the anodic peak potential against pH (b). Figure S4 CVs of the 0.48 μg PtNi/C modified GCE in 0.1 M BR buffer solution (pH = 2.0) containing 0.5 mM caffeic acid at scan rates from 20 to 200 mV s−1 (a) and the plots of anodic and cathodic peak currents to the scan rates (b). Figure S5 The CVs of the 0.48 μg PtNi/C electrode (a) and Pt/C electrode (c) in 0.5 mM CA after various numbers of potential cycles (1–4000 cycles) at a scan rate of 100 mV s−1. The oxidation peak currents of 0.48 μg PtNi/C electrode (b) and Pt/C electrode (d) vs number of cycles. Figure S6 (a) TEM images, (b) HAADF-STEM images and (c) STEM-EDS elemental mapping of Pt-Ni jagged-like nanowires after subjecting the sample to potential cycling for 4000 cycles between 0.1–1.0 V in 0.1 M BR buffer solution electrolyte at 100 mV s−1. Figure S7 Relative analytical response (Ipa/Ip) of PtNi/C electrode in 0.1 M BR buffer solution (pH = 2.0) containing 0.5 mM caffeic acid in presence of different interfering species: 10 mM tannic acid, resveratrol, tartaric acid, gallic acid, citric acid, P-coumaric acid, succinic acid, and malic acid. Table S1 Comparison of the linear range and detection limit between the proposed method and other reported detection methods for caffeic acid. Table S2 Determination of CA in red wine samples (DOCX 2679 kb) [file 11671_2018_2839_MOESM1_ESM.docx]

**Supplementary Information**

**Ultra stable electrochemical sensor for detection of caffeic acid based on platinum and nickel jagged-like nanowires**

Jin Wang^a^, Beibei Yang^a^, Fei Gao^a^, Pingping Song^a^, Lei Li^*b^, Yangping Zhang^a^, Cheng Lu,^*c^, M. Cynthia Goh,^*c^ and Yukou Du^*a^

*^a^College of Chemistry, Chemical Engineering and Materials Science, Soochow University, Suzhou 215123, PR China.*

*^b^College of Biological, Chemical Sciences and Engineering, Jiaxing University, Jiaxing, Zhejiang 314001, China.*

*^c^Department of Chemistry, Department of Materials Science and Engineering, Institute of Medical Science, University of Toronto. Toronto, ON M5S 3H6, Canada.*

^*^Corresponding authors: duyk@suda.edu.cn, [leili@mail.zjxu.edu.cn](mailto:leili@mail.zjxu.edu.cn), clu@chem.utoronto.ca and cgoh@chem.utoronto.ca.





Fig. S1 XRD patterns of PtNi jagged-like nanowires.


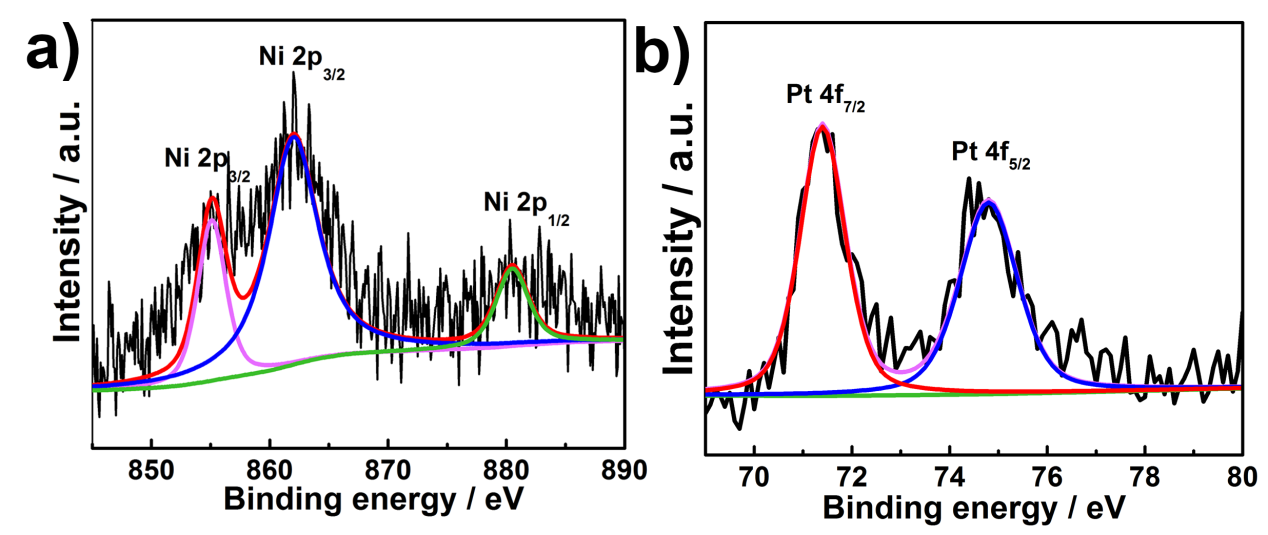


Fig. S2 (a) Pt 4f and (b) Ni 2p deconvoluted XPS spectras of PtNi jagged-like nanowires.


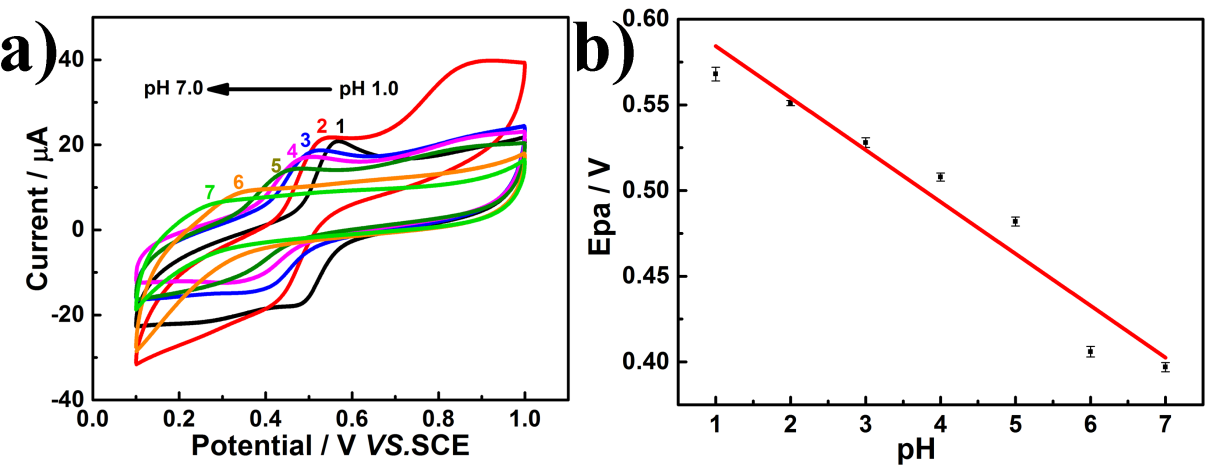


Fig. S3 The CVs of 0.48 μg PtNi-C modified GCE in 0.1 M BR buffer solution containing 0.5 mM caffeic acid at pH ranging from 1.0 to 7.0 (a) and the plots of the anodic peak potential against pH (b).


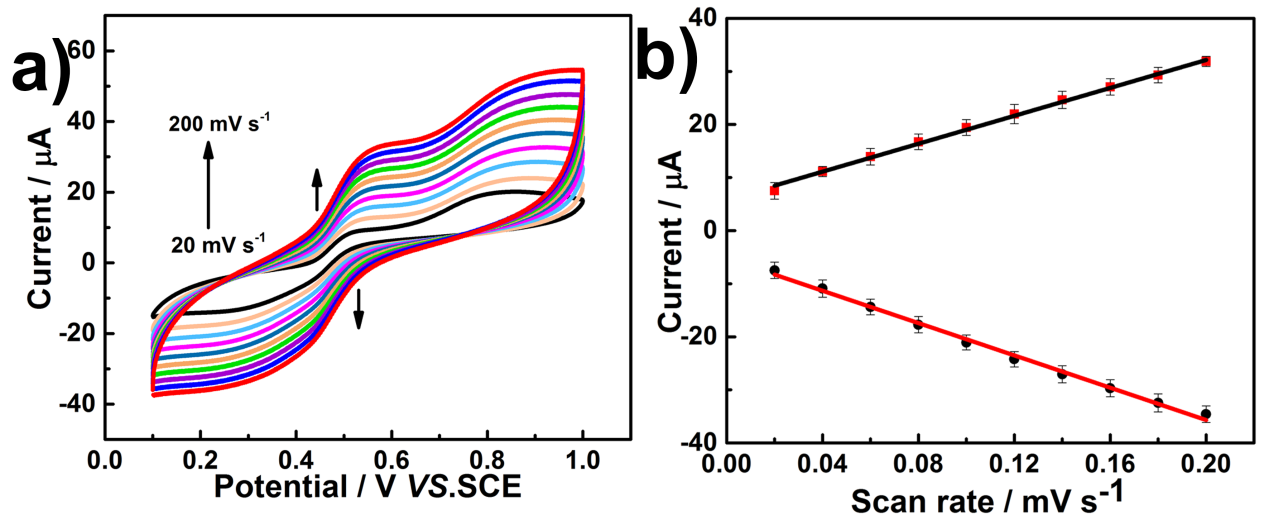


Fig. S4 CVs of the 0.48 μg PtNi-C modified GCE in 0.1 M BR buffer solution (pH = 2.0) containing 0.5 mM caffeic acid at scan rates from 20 to 200 mV s^-1^ (a) and the plots of anodic and cathodic peak currents to the scan rates (b).


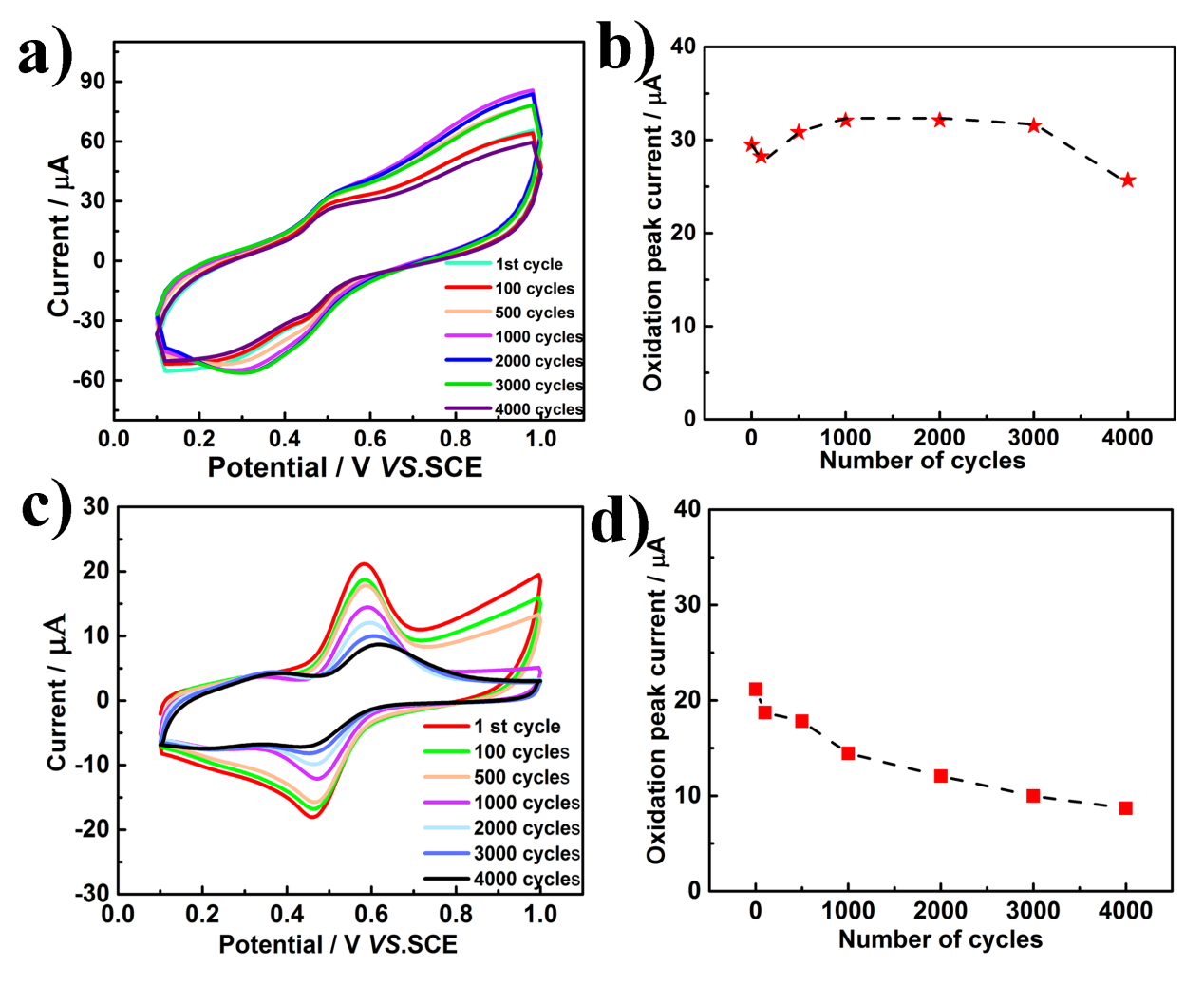


Fig. S5 The CVs of the 0.48 μg PtNi/C electrode (a) and Pt/C electrode (c) in 0.5 mM CA after various numbers of potential cycles (1-4000 cycles) at a scan rate of 100 mV s^−1^. The oxidation peak currents of 0.48 μg PtNi/C electrode (b) and Pt/C electrode (d) vs number of cycles.


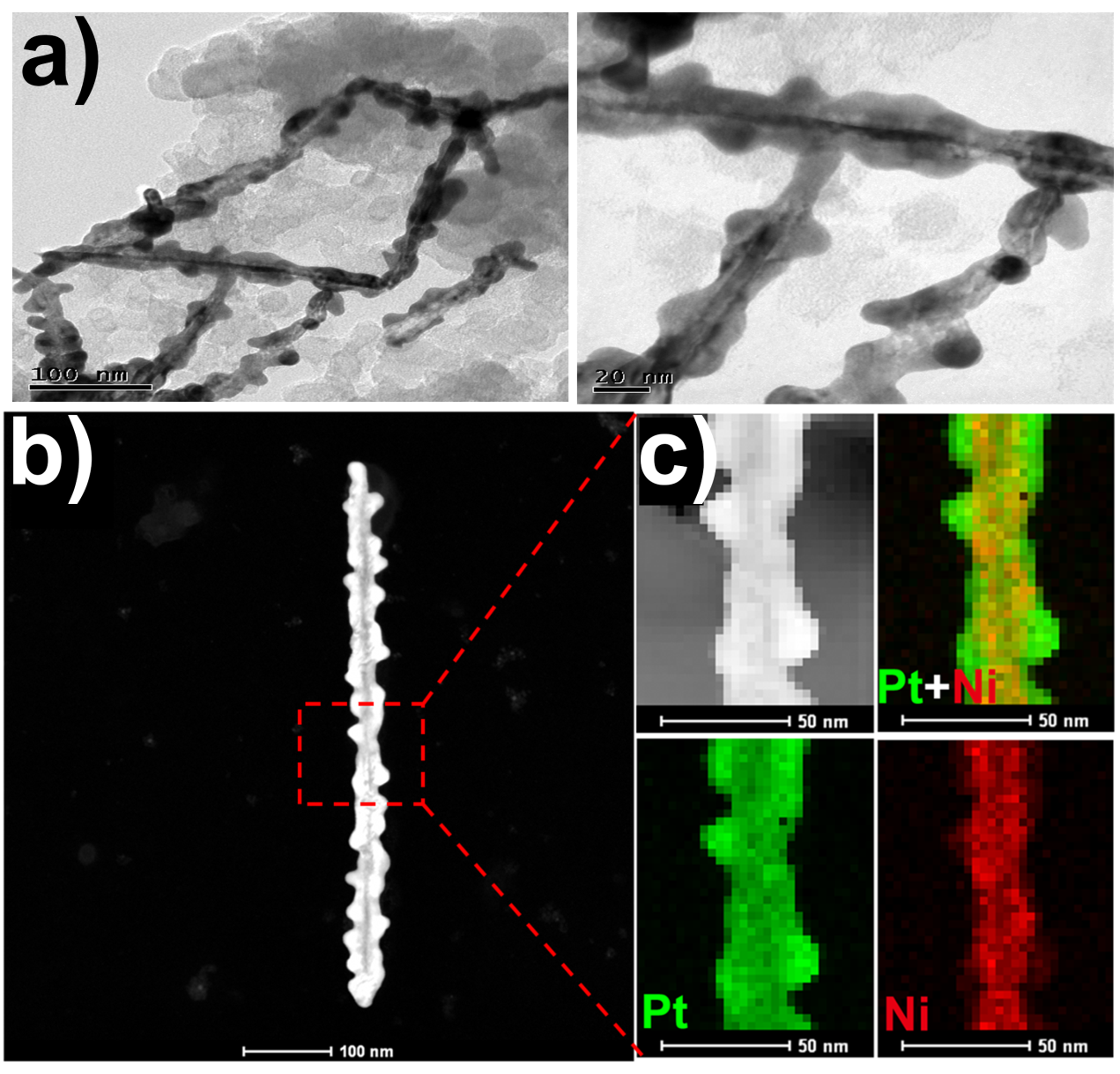


Fig. S6 (a) TEM images, (b) HAADF-STEM images and (c) STEM-EDS elemental mapping of Pt-Ni jagged-like nanowires after subjecting the sample to potential cycling for 4000 cycles between 0.1 - 1.0 V in 0.1 M BR buffer solution electrolyte at 100 mV s^-1^.





Fig. S7 Relative analytical response (I_pa_/I_p_) of PtNi-C electrode in 0.1 M BR buffer solution (pH = 2.0) containing 0.5 mM caffeic acid in presence of different interfering species: 10 mM Tannic acid, Resveratrol, Tartaric acid, Gallic acid, Citric acid, P-coumaric acid, Succinic acid and Malic acid.

Table S1 Comparison of the linear range and detection limit between the proposed method and other reported detection methods for caffeic acid

| Electrode | Technique | Linear range (μM) | Detection limit (μM) | Ref |
| --- | --- | --- | --- | --- |
| LDH/GCE | DPV | 7 - 180 | 2.6 | [1] |
| CPE | SWV | 20 - 200 | 2 | [2] |
| Poly/GCE | LSV | 9 - 40 | 3.91 | [3] |
| G-quadruplex/hemin DNAzyme | Fluorometry | 2 - 350 | 0.2 | [4] |
| CdTe QDs | Fluorometry | 3.71 – 111.8 | 1.2 | [5] |
| PtNi-C/GCE | DPV | 0.75 – 591.78 | 0.5 | This work |

Table S2 Determination of CA in red wine samples

| Red Wine samples | Added CA  (μM L^-1^) | Found CA  (μM L^-1^) | | | RSD  (%) | Recovery  (%) |
| --- | --- | --- | --- | --- | --- | --- |
| 1 | 50 | 51.24 | 51.37 | 51.32 | 0.13 | 100.52 |
| 2 | 100 | 101.85 | 101.64 | 102.23 | 0.29 | 100.19 |
| 3 | 200 | 205.17 | 205.14 | 204.95 | 0.06 | 100.61 |

**References**

1. Kahl M, Golden T. D, (2014) Electrochemical determination of phenolic acids at a Zn/Al layered double hydroxide film modified glassy carbon electrode. Electroanalysis 26: 1664 1670.

2. Fernandes S.C, Oliveira I. R. W. Z, Vieira I. C, (2007) A green bean homogenate immobilized on chemically crosslinked chitin for determination of caffeic acid in white wine, Enzyme and Microbial Technology 40:661 668.

3. Santos D. P, Bergamini M. F, Fogg A. G, Zanoni M. V. B, (2005) Application of a glassy carbon electrode modified with poly(glutamic acid) in caffeic acid determination, Microchimica Acta 151: 127 134.

4. Cai N, Li Y, Chen S, Su X, (2016) A fluorometric assay platform for caffeic acid detection based on the G-quadruplex/hemin DNAzyme, Analyst 141: 4456 4462.

5. Fan X, Liu S, He Y, (2011) Study on the interaction of CdTe quantum dots with coumaric acid and caffeic acid based on fluorescence teversible tune, Colloid Surface B 88: 23-30.
